# Novel variant c.7795-1G>A of COL7A1 gene in a 12-month-old female child with recessive dystrophic epidermolysis bullosa treated with dupilumab

**DOI:** 10.1016/j.jdcr.2024.06.003

**Published:** 2024-06-09

**Authors:** Francesca Caroppo, Fortunato Cassalia, Elisa Milan, Anna Belloni Fortina

**Affiliations:** a Dermatology Unit, Department of Medicine, University of Padua, Padua, Italy; b Department of Women and Children’s Health, Pediatric Dermatology Regional Center, University of Padua, Padua, Italy; c European Reference Network for Rare Skin Disorders- ERN Skin, Department of Women's and Children's Health, University of Padua, Padua, Italy

**Keywords:** congenital diseases, DEB, dystrophic epidermolysis bullosa, EB, epidermolysis bullosa, genetic diseases, mutation, rare skin diseases

## Introduction

Epidermolysis bullosa (EB) is a group of inherited disorders characterized by skin fragility and blistering in response to minor trauma. The prevalence of EB in the United States is estimated to be 11.1 cases per million live births. Classification of EB, based on evolving genetic knowledge, is critical to understanding and managing these conditions.^1^ Recessive dystrophic EB (RDEB) is a very rare disease with an incidence of 3.05 × 10^6^ people in the United States, and it is the most severe subtype of EB. It is caused by mutations in the COL7A1 gene. RDEB is characterized by widespread blistering, painful and slow-healing wounds, and scarring. Systemic manifestations include dental abnormalities, nail dystrophy, and corneal erosions.^1^^,^^2^

We describe a case of a 12-month-old female child with a novel mutation of COL7A1 gene causing RDEB, also reporting data about efficacy and safety of treatment with dupilumab.

## Case report

**Fig 1:**
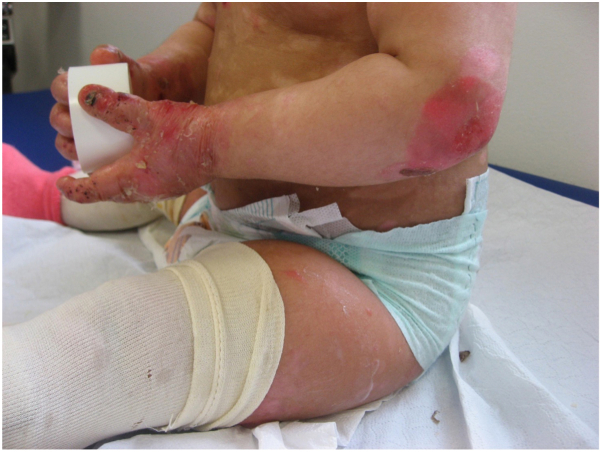
First clinical evaluation: deepithelialized areas, blisters, and excoriations on the elbows and distal extremities of the upper limbs.

**Fig 2:**
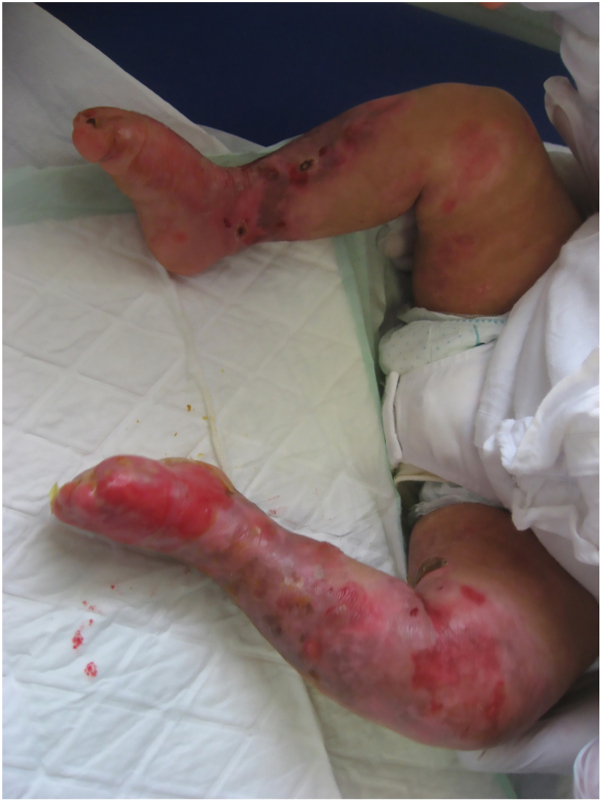
First clinical evaluation: deepithelialized areas, blisters, and excoriations on the lower limbs.

We present the case of a 12-month-old female child who was referred to our Pediatric Dermatology Regional Center of the University of Padua because of recurrent blisters and boils predominantly on the upper and lower limbs since birth. The child had severe pruritus and excoriations (Figs 1 and 2). The family history was negative for dermatological and genetic diseases. Given the presentation and clinical suspicion of a genetic blistering disease, we performed a skin biopsy and genetic testing.

Histologic examination showed hyperkeratosis and mild acanthosis with a peri-vascular mononuclear inflammatory infiltrate in the papillary dermis. At the dermoepidermal junction, there was a focal separation. Immunofluorescence test showed a cleavage plane located below the dense lamina of the dermoepidermal junction against which anti-type IV collagen antibodies react.

Genetic analysis identified the c.7795-1G>A variant within the COL7A1 gene, confirming the diagnosis of RDEB. This specific mutation had not been previously reported in the literature in patients with RDEB and is absent in the general population genome aggregation database.

**Fig 3:**
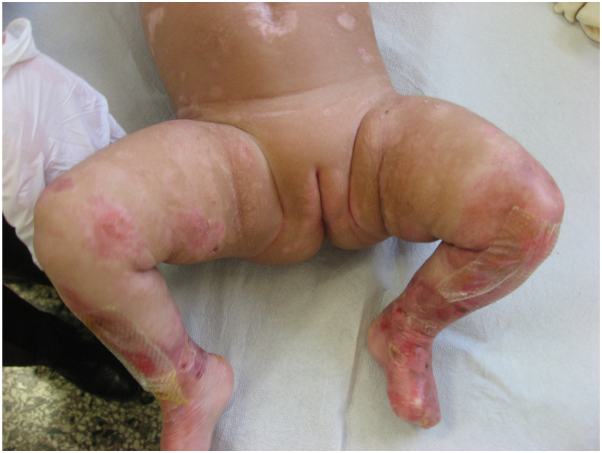
Clinical follow-up 3 months after starting dupilumab therapy.

Our initial therapeutic approach included regular skin assessments, wound care based on local antiseptics and soft silicone wound contact layer dressings, and symptomatic treatment with systemic antihistamines (oxatomide at the dosage of 0.5 mg/kg/die). However, the itching was poorly controlled with an numeric rating score of 9 and the skin lesions worsened. Therefore, in light of the previously reported cases of EB successfully treated with dupilumab,^3^, ^4^, ^5^, ^6^ we decided to start dupilumab at the dosage of 200 mg administered subcutaneously, followed by 200 mg every 4 weeks, with good results and no adverse events, observing a significant clinical improvement after 3 months (Fig 3) and a substantial reduction of pruritus with an numeric rating score of 2.

## Discussion

RDEB is caused by mutations in the COL7A1 gene, which encodes type VII collagen, an essential protein for skin integrity. RDEB is clinically characterized by severe mucocutaneous fragility leading to widespread blistering and scarring, a more severe manifestation than some other EB subtypes.^2^

To the best of our knowledge, the c.7795-1G>A mutation of COL7A1 gene observed in this case was never described before in patients with dystrophic EB (DEB) and is absent in the general population genome aggregation database.

Inflammation is an essential pathogenic feature in patients with RDEB; in response to mechanical injury, keratinocytes release several proinflammatory cytokines. These could be released into the systemic circulation and could mediate the recruitment of inflammatory cells and development of T helper 17 and T helper 2 cells at the injury site.^7^

The pathogenetic mechanisms involved in pruritus in DEB are still unclear; they may be related to a complex interaction involving barrier dysfunction, keratinocytes, cutaneous nerve fibers, and cytokines. Keratinocytes and immune cells express receptors for various itch mediators, whose activation leads, in turn, to the release of further pruritogens (such as interleukin [IL]-4, IL-13, and IL-31). Itching could be also related to a dysregulation of itch-related mediators and cytokine and to the wound healing process.^7^^,^^8^

Use of dupilumab is a fully human monoclonal antibody targeting the α subunit of IL-4 receptor, thus inhibiting IL-4 and IL-13 cytokines effects. Some authors reported an effective use of dupilumab in patients with junctional, simplex, and DEB^9^^,^^10^; therefore, we decided to start dupilumab at the dosage approved for atopic dermatitis in children, observing good clinical results and no adverse events.

Our case suggests that DEB could be driven by T helper 2 immune mechanisms and that dupilumab could be represent a therapeutic option, also in pediatric patients. Future larger studies are certainly needed to better understand and confirm the mechanisms of action of dupilumab in EB.

## Conflicts of interest

Caroppo has been a consultant for Leo Pharma, Sanofi Genzyme, AbbVie, Hollister, Amgen. Belloni Fortina has been a consultant for Almirall, Amgen, Sanofi Genzyme, Pfizer, AbbVie, LeoPharma, Unifarco, Novartis, Eli Lilly. Cassalia, Milan have no conflicts of interest.
